# Targeted regions sequencing identified four novel *PNPLA1* mutations in two Chinese families with autosomal recessive congenital ichthyosis

**DOI:** 10.1002/mgg3.1076

**Published:** 2019-12-13

**Authors:** Liangshan Li, Wenmiao Liu, Yinglei Xu, Miaomiao Li, Qian Tang, Bo Yu, Renmei Cai, Shiguo Liu

**Affiliations:** ^1^ Medical Genetic Department The Affiliated Hospital of Qingdao University Qingdao China; ^2^ Prenatal Diagnosis Center The Affiliated Hospital of Qingdao University Qingdao China; ^3^ Department of Clinical Laboratory Medical College of Qingdao University Qingdao China; ^4^ Dermatological department The Affiliated Hospital of Qingdao University Qingdao China; ^5^ Prenatal Diagnosis Center Qingdao Municipal Hospital Qingdao China

**Keywords:** autosomal recessive congenital ichthyosis (ARCI), genetic analyses, *PNPLA1*, Sanger sequencing, targeted regions sequencing (TRS)

## Abstract

**Background:**

Autosomal recessive congenital ichthyosis (ARCI) is a rare genetically heterogeneous cutaneous disease predominantly characterized by erythroderma, generalized abnormal scaling of the whole body and a collodion membrane at birth. Numerous causative genes have been demonstrated to be responsible for ARCI including *PNPLA1* which can cause ARCI type 10. The objectives of this study are to describe clinical features of three ARCI patients from two Chinese unrelated families and to identify the underlying causative mutations.

**Methods:**

Genomic DNA was extracted from peripheral venous blood obtained from the two Chinese ARCI families in Shandong province. Subsequently, targeted regions sequencing (TRS) followed by Sanger sequencing was conducted to identify and validate the likely pathogenic mutations of the ARCI families.

**Results:**

Genetic analyses revealed four novel *PNPLA1* variants that are predicted to be probably to lead to ARCI in three patients of two families. Patient 1 in one family was in compound heterozygous status for c.604delC/p.Arg202Glyfs*27 and c.820dupC/p.Arg274Profs*15, whereas c.738_742delinsCCCACAGATCCTGC/ p.Gly247_Tyr248delinsProGlnIleLeuHis, and c.816dupC/p.Arg274Profs*15 were found in patient 2 and 3 of the other family. In addition, these variants cosegregate in the two pedigrees and are all within highly conserved regions of the PNPLA1 protein, which indicate that the four mutations are likely pathogenic.

**Conclusion:**

Our findings not only broaden the mutational spectrum of PNPLA1, but also contribute to establishing genotype–phenotype correlations for different forms of ARCI.

## Graphical abstract

Genetic analyses in the present study revealed four novel PNPLA1 variants that are predicted to be probably to lead to ARCI in three patients of two families. In addition, these variants co segregate in the two pedigrees and are all within highly conserved regions of the PNPLA1 protein, which indicate that the four mutations are likely pathogenic.

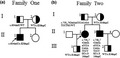

## 1 INTRODUCTION

As a clinically and genetically heterogeneous skin disorder, ichthyoses are characterized by extensively dry and scaly skin almost covering the whole body and sometimes with erythroderma and a collodion membrane (Takeichi & Akiyama, 2016). It has been divided into two categories, syndromic and nonsyndromic ichthyoses, whereas the latter whose symptoms appear only in the skin, can be classified into common ichthyoses, autosomal recessive congenital ichthyosis (ARCI), keratinopathic ichthyosis (KPI), and other forms of ichthyoses (Oji et al., 2010; Takeichi & Akiyama, 2016). With an approximate prevalence of 1:200,000, ARCI is clinically classified as three primary subtypes including congenital ichthyosiform erythroderma (CIE, OMIM 242100), lamellar ichthyosis (LI, OMIM 242300), and the less common harlequin ichthyosis (HI, OMIM 242500) which is more severe than the other two subgroups (Esperon‐Moldes et al., 2019; Karim, Murtaza, & Naeem, 2017). ARCI is transmitted in an autosomal recessive pattern with common clinical signs of generalized scales sometimes accompanied by erythroderma or a collodion membrane, although the phenotypes of affected patients may be greatly variable (Simpson et al., 2019).

To date, Online Mendelian Inheritance in Man (OMIM) database has described fifteen genetic ARCI types (ARCI type 1 to type 14) that are associated with *LIPN* (OMIM 613924), *CASP14* (OMIM 605848), *CYP4F22* (OMIM 611495), *ABCA12* (OMIM 607800), *NIPAL4* (OMIM 609383), *ALOXE3* (OMIM 607206), *SULT2B1* (OMIM 604125), *ABCA12* (OMIM 607800), *SDR9C7* (OMIM 609769), *CERS3* (OMIM 615276), *ALOX12B* (OMIM 603741), *ST14* (OMIM 606797), *TGM1* (OMIM 190195), *PNPLA1* (OMIM 612121) (Heinz et al., 2017; Karim, Durbin‐Johnson, et al., 2019; Karim, Ullah, Murtaza, & Naeem, 2019), among which *PNPLA1* (patatin like phospholipase domain containing 1), the causative gene of ARCI type 10 (OMIM 615024), spans over 71 kb in size at chromosome 6p21.31 and comprises 11 exons.

The corresponding coding protein PNPLA1 has 532 amino acids in length (NM_001145717.1) and is one of nine members of the PNPLA protein family that share a common patatin‐like domain (Wilson, Gardner, Lambie, Commans, & Crowther, 2006). PNPLA1 consists of an entire patatin domain (residues 16–185) at the N‐ terminus and a proline‐rich C‐terminal domain (residues 326–451) where a hydrophobic domain ranging from Leu335 to Ser417 is located (PA et al., 2013). In addition, PNPLA1, mainly expressed in the keratinocytes of epidermal granular layer (PA et al., 2013), serves a significant role in the glycerophospholipid metabolism of the cutaneous barrier (Esperon‐Moldes et al., 2019). A majority of *PNPLA1* mutations usually involve in the N‐terminal highly conserved patatin domain named mutational “hot‐spots” region and most ARCI cases carry nonsense or missense variants (Diociaiuti et al., 2018; Karim, Ullah, et al., 2019).

With the advantages of high throughput, high cost‐effectiveness, fast speed and high accuracy, the targeted regions sequencing (TRS) technology is widely used in the auxiliary diagnoses and classifications of genetic diseases that have several disease‐causing genes. Additionally, TRS aims to sequence the pathogenic genes of certain specific diseases, thus reducing the costs greatly. The predominant objectives of this study are to describe the detailed clinical features of three Chinese ARCI patients from two unrelated families and to identify the underlying likely pathogenic mutations responsible for the ichthyoses phenotypes, which could facilitate the genetic counseling for the ARCI family, therefore further improving the quality of the population.

## 2 MATERIALS AND METHODS

### 2.1 Ethical compliance

This study was approved by the Ethics Committee of the Affiliated Hospital of Qingdao University.

### 2.2 Patients

**Figure 1:**
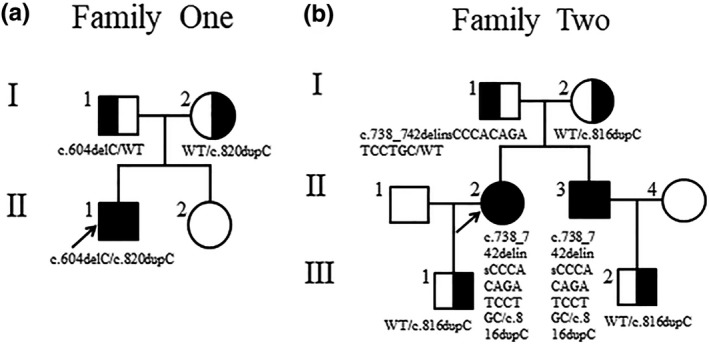
Pedigrees of two Chinese ARCI families including genotype information. (a) Family one. (b) Family two. The arrowheads denote the probands, filled symbols represent the patients, and half‐filled symbols indicate the carriers

In this study, we investigated three ARCI patients (Shandong, China) from two unrelated Chinese non‐consanguineous families (Figure 1). P1 (7 years old; II1, Figure 1a), P2 (61 years old; II2, Figure 1b), and P3 (56 years old, younger brother of P2; II3, Figure 1b) were diagnosed with ARCI type 10 based on clinical manifestations combined by the results of molecular genetic detection. However, none of their other family members exhibited similar symptoms. Blood samples were collected from the patients, their family members and 100 unrelated individuals after having obtained written informed consent from them.

### 2.3 DNA extraction

The genomic DNA was extracted from peripheral venous blood using a Qiagen DNA extraction kit, strictly following the manufacturer's protocol. The concentration of the genomic DNA was measured by a spectrophotomer (Thermo Fisher Scientific Oy Ratastie 2, FI‐01620 Vantaa, Finland).

### 2.4 TRS for mutation detection

Molecular analyses for all exons of 40 known genes associated with ichthyoses including *PNPLA1, ABHD5, ALDH3A2, ALOX12B, ALOXE3, AP1S1, C7ORF11, CDSN, CLDN1, CSTA, EBP, ERCC2, ERCC3, FLG, GBA, KRT1, KRT10, KRT2, LOR, MBTPS2, NIPAL4, NSDHL, PEX7, PHYH, POMP, SLC27A4, SNAP29, SRD5A3, TGM1, VPS33B, LIPN, CYP4F22, GJB2, GJB3, GJB6, GJB4, ABCA12, SPINK5, STS,* and *ST14* were performed using TRS technology on the patients.

The DNA was quantified with Nanodrop 2000 (Thermal Fisher Scientific). DNA fragments of 100–700 bp were obtained by random interruption of qualified genomic DNA using a Covaris crusher and then fragments with sizes ranging from 350 to 450 bp and those including the adapter sequences were selected for the DNA libraries preparation. The biotinylated capture probes (80–120‐mer) were hybridized with DNA libraries under certain conditions. The magnetic beads modified by streptavidin covalently combined with the biotin labeled probes to capture the target genes. Subsequently, the magnetic beads carrying the target genes were absorbed by magnetic frame, washed off and purified for the enrichment of the target genes. Finally, The enriched libraries were sequenced on an Illumina NextSeq 500 sequencer for paired‐end reads of 150 bp.

Following sequencing, low‐quality variations were filtered out using a quality score ≥20 and BWA was used to align the clean reads to the reference human genome (hg19). The identified SNPs and InDels were annotated using the Exome‐assistant program (http://122.228.158.106/exomeassistant). The frequency >0.02 of SNPs and InDels in HapMap samples, 1,000 Genome, ESP6500,ExAC_ALL and ExAC_EAS were removed. The variants were evaluated by several bioinformatics software programs to predict their pathogenicity.

### 2.5 Sanger sequencing validation

The *PNPLA1* variants of the patients identified by NextSeq 500 sequencing were validated by Sanger sequencing. Genomic DNA from all available family members were obtained for Sanger sequencing. Amplified polymerase chain reaction (PCR) products were analyzed by gel electrophoresis, then purified and sequenced on an ABI PRISM 3730 genetic analyzer (Applied Biosystems; Thermo Fisher Scientific, Inc.) using the terminator cycle sequencing method. Loci of the mutations were identified through the comparison of DNA sequences with the reference sequences on the National Center Biotechnology Information (NCBI) website (https://www.ncbi.nlm.nih.gov/).

## 3 RESULTS

### 3.1 Clinical manifestations

**Figure 2:**
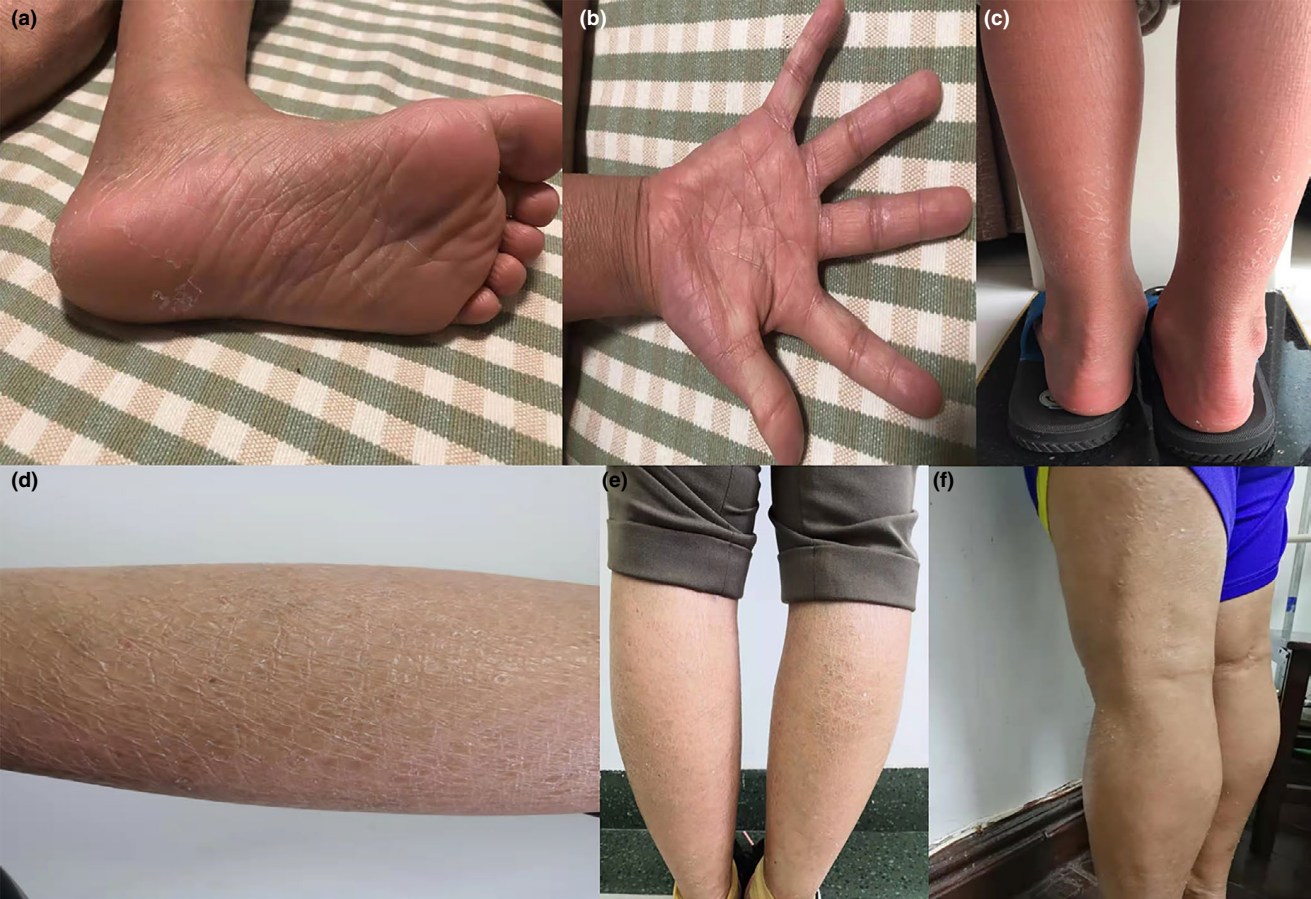
Clinical characteristics of the ARCI patients. (a–c) P1 exhibits large whitish dry scales, sole and palmar hyper linearity, desquamation on the soles with erythroderma. (d, e) P2 manifests dry scaly skin. (f) P3 shows generalized scaling and fine whitish scales without erythema

P1 (Figure 2a–c) was born as a severe collodion baby with erythroderma. Whitish plate‐like and dry scales can be seen all over the body accompanied by pruritus except for the face which usually exacerbate in spring and autumn. In addition, he also presents with increased cerumen, palmoplantar hyperlinearity and abnormal desquamation on the soles. However, the phenotypic features of P2 (Figure 2d,e) and P3 (Figure 2f) are similar and are milder than P1. Both of them began to develop skin lesions three months after they were born without a collodion membrane covering their bodies or apparent erythroderma. They manifest typical phenotypes of ichthyoses as generalized dry, fine and whitish scales which are more severe primarily at the turn of the seasons, but never with pruritus. Mild palmoplantar hyperlinearity can be also observed. No other members in their families have identical or similar signs and symptoms.

### 3.2 Genetic analyses

**Figure 3:**
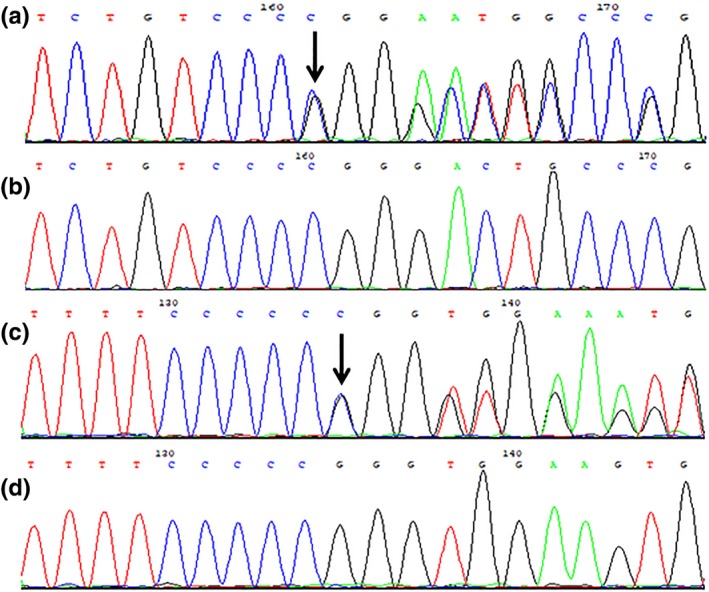
Sequence chromatograms of family one. (a) The deletion mutation c.604delC/p.Arg202Glyfs*27 in exon 4, (b) homozygous wild type, (c) the duplication mutation c.820dupC/p.Arg274Profs*15 in exon 6, and (d) homozygous wild type. The black arrowheads denote the loci of the mutations

Mutational analyses of P1 revealed two novel frameshift variants in *PNPLA1* (transcript variant 3, NM_001145717.1), c.604delC/p.Arg202Glyfs*27 in exon 4 (Figure 3a) is a deletion mutation, whereas the other variant c.820dupC/p.Arg274Profs*15 in exon 6 (Figure 3c) is a duplication mutation, inherited from the father (I1, Figure 1a) and mother (I2, Figure 1a), respectively. The c.604delC mutation is predicted to be disease causing by MutationTaster (://www.mutationtaster.org/) with a score of 1.

**Figure 4:**
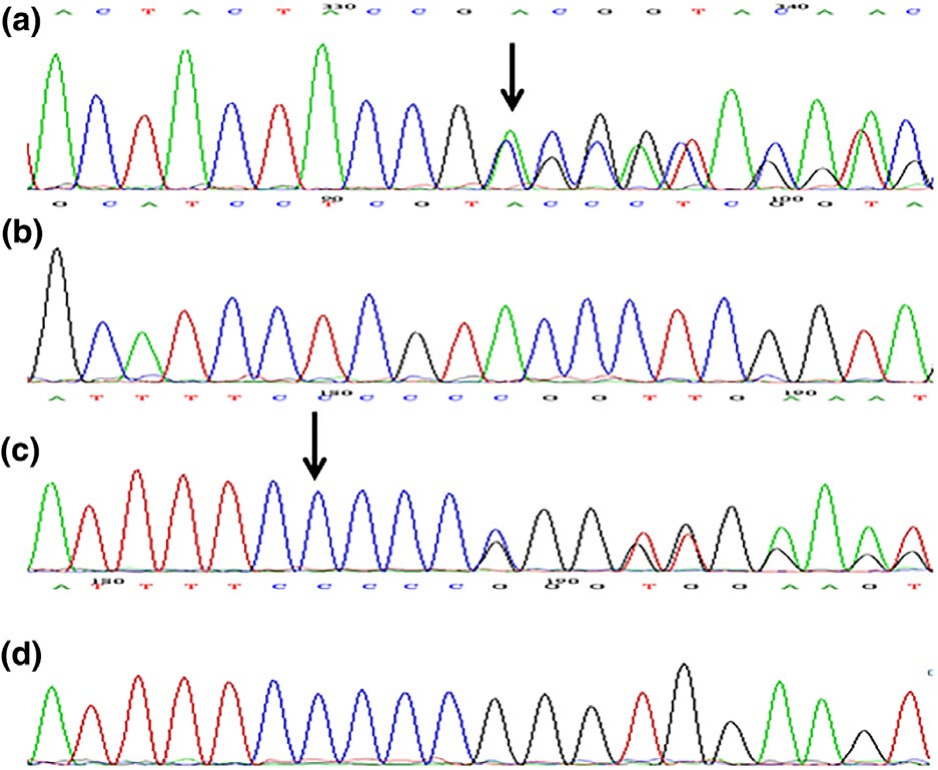
Sequence chromatograms of family two. (a) The in‐frame mutation c.738_742delinsCCCACAGATCCTGC/p.Gly247_Tyr248delinsProGlnIleLeuHis in exon 5, (b) homozygous wild type, (c) the duplication mutation c.816dupC/p.Arg274Profs*15 in exon 6 and (d) homozygous wild type. The black arrowheads denote the loci of the variants. The black arrowheads denote the loci of the mutations

Two novel compound heterozygous variants c.738_742delinsCCCACAGATCCTGC/p.Gly247_Tyr248delinsProGlnIleLeuHis in exon 5 (Figure 4a) and c.816dupC/p.Arg274Profs*15 in exon 6 (Figure 4c) were identified in P2 and P3, of which the former is an in‐frame mutation, whereas the latter is a duplication one. The variant c.738_742delinsCCCACAGATCCTGC is classified as deleterious by PROVEAN (Protein Variation Effect Analyzer, http://provean.jcvi.org/seq_submit.php) with a score of −22.145 (cutoff = −2.5). The father (I1, Figure 1b) is found in heterozygous status of p.Gly247_Tyr248delinsProGlnIleLeuHis, however, the mother (I2, Figure 1b) and their sons (III1 and III2, Figure 1b) are all heterozygotes for p.Arg274Profs*15 mutation. In addition, no other pathogenic or likely pathogenic variants were identified in any of the 40 sequenced ichthyosis‐related genes.

All mutations are not annotated in HGMD, ESP6500siv2_ALL, 1000g2015aug_ALL, Clinvar, ExAC, and dbSNP147 databases, and are absent in 100 healthy controls (Figures 3b,d and 4b,d). cosegregation analyses indicate that the inheritance mode of this disease in the families is consistent with the autosomal recessive pattern of ARCI type 10 and the four novel variants are responsible for the ARCI presentations of the three patients, thus suggesting the *PNPLA1* mutations are likely pathogenic.

### 3.3 Bioinformatic analyses of the *PNPLA1* mutations

**Figure 5:**
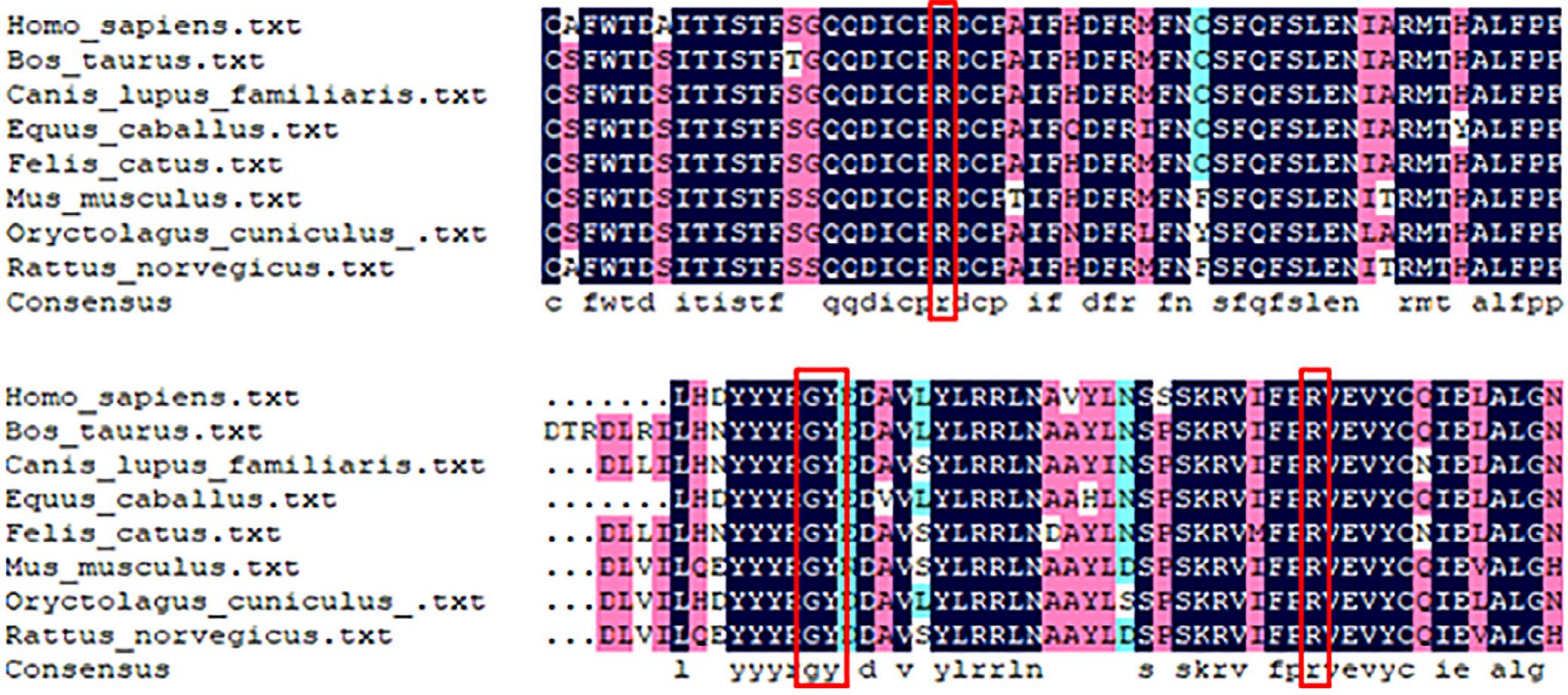
Comparison of amino acid sequences of PNPLA1 protein across different species. The red rectangular frames indicate the locations of R202, G247, Y248 and R274

Protein sequences of various species including *Homo sapiens*, *Bos taurus*, *Canis lupus familiaris*, *Equus caballus*, *Felis catus*, *Mus musculus*, *Oryctolagus cuniculus,* and *Rattus norvegicus* were obtained from NCBI website. Multiple sequence alignment of the PNPLA1 protein among these species was carried out using DNAMAN software and the results suggested that R202, G247, Y248, and R274 were all localized within the highly conserved domain of PNPLA1, but outside the core patatin domain (residues 16–185) (Figure 5).

## 4 DISCUSSION

In this study, we identified four previously unknown *PNPLA1* mutations (three frameshift mutations and one in‐frame mutation) through TRS in combination with Sanger sequencing in two unrelated ARCI type 10 families of Chinese origin. The frameshift mutations c.604delC, c.820dupC, and c.816dupC that are classified as likely pathogenic according to American College of Medical Genetics and Genomics (ACMG) guidelines with the criteria of PVS1 + PM2 + PM3 can result in PNPLA1 protein truncation or nonsense‐mediated RNA decay with loss of protein expression from this allele which may have damaging impacts on the normal structure and function of the corresponding protein. Additionally, the three mutations can lead to the loss of PNPLA1 C‐terminal region to a large extent, which is essential for protein activity (Karim, Ullah, et al., 2019). The in‐frame variant c.738_742delinsCCCACAGATCCTGC is identified as a probably pathogenic mutation based on protein prediction tool and ACMG guidelines with the classification criteria of PM2 + PM3 + PM4. Moreover, the four novel variants co‐segregate in these families supported by the finding that they were not found in 100 Chinese healthy individuals.

As a subgroup of nonsyndromic ichthyoses, ARCI is a general term for a group of rare genetic cornification disorders, ranging from relatively mild to very severe in the severity of the disease, sometimes even life‐threatening (Bastaki et al., 2017). ARCI can be divided into six forms on the basis of diverse clinical manifestations involving congenital ichthyosiform erythroderma (CIE), lamellar ichthyosis (LI), and harlequin ichthyosis (HI), Self‐healing collodion baby (SHCB), acral self‐healing collodion baby (acral SHCB), bathing suit ichthyosis (BSI) (Simpson et al., 2019). The frequent clinical phenotypes comprise a collodion membrane at birth, generalized fine or plate‐like dry scales with whitish, dark gray or brown in color, erythroderma, and palmoplantar hyperlinearity. ARCI patients may also exhibit palmoplantar keratoderma (PPK), swollen hands and feet, anhidrosis, alopecia, nail abnormalities, and ectropion (Boyden et al., 2017).

Autosomal recessive congenital ichthyosis is inherited in an autosomal recessive trait, therefore patients are homozygous or compound heterozygous for pathogenic bi‐allelic mutations. In addition, ARCI has been classified into fifteen genetic forms based on their causative genes including *PNPLA1*, *LIPN*, *CASP14*, *CYP4F22*, *ABCA12*, *NIPAL4*, *ALOXE3*, *SULT2B1*, *ABCA12*, *SDR9C7*, *CERS3*, *ALOX12B*, *ST14*, *TGM1* (Fachal et al., 2014), of which the underlying pathogenic gene responsible for ARCI type 7 has not been defined. Although the clinical features of ARCI patients resulting from *PNPLA1* mutations vary, the degree of severity is milder than other subtypes of ARCI caused by mutated *TGM1* and *ABCA12* (Zimmer et al., 2017).

*PNPLA1,* associated with ARCI type 10 phenotypes, was first described to be a causative gene for *ARCI* in humans and dogs by Grall et al. (2012). *PNPLA1* has been demonstrated to be located on chromosome 6p21.31 and possesses genomic DNA of 71,433 bp. The protein encoded by *PNPLA1* is crucial for generating omega‐O‐acylceramides (ω‐O‐AcylCers) in the maintenance of cutaneous integrity and barrier function, and belongs to the mammalian PNPLA family that contains a highly conserved core patatin domain which is ubiquitous in potato tubers (Grond et al., 2017; Kienesberger, Oberer, Lass, & Zechner, 2009; Zimmer et al., 2017). PNPLA family (PNPLA1–9) is one of the patatin superfamily members and serves a key role in diverse aspects of lipid metabolism and signal pathway involving triglyceride lipase, hydrolase, and transacetylase activities (Dokmeci‐Emre et al., 2017; Pichery et al., 2017). PNPLA1 mutations have been identified in approximately of 3% patients clinically diagnosed with ARCI (Zimmer et al., 2017). To date, the definite associations between genotype and the phenotype of ARCI have not been clearly elucidated due to clinical heterogeneity.

Musharraf Jelani *et al.* described a Pakistani family affected with ARCI, the first ichthyoses case caused by defective *PNPLA1* in Asia. A homozygous missense mutation c.387C>A (p.Asp129Glu) lying in the highly conserved patatin domain was identified in the patients who presented with a collodion membrane at birth and fine whitish scales covered most of the body surface. Simultaneously, this novel variant was predicted to be damaging in silico analyses (Lee et al., 2016). In 2017, a Turkish ARCI family who carried a novel homozygous deletion mutation c.733_735delTAC (p.Tyr245del) in exon 5 of *PNPLA1*was reported, with the clinical characteristics of erythema, small whitish and light brown scales accompanied by pruritus, PPK, toenail dystrophy and distal onycholysis. This variant was localized at the extended patatin domain (amino acids 1–288) between core patatin domain and proline‐rich region of PNPLA1, and was evaluated as deleterious by several prediction programs (Dokmeci‐Emre et al., 2017). Consistent with the mutant locus, four novel *PNPLA1* mutations identified in this study are all located at the extended patatin domain but outside the core patatin domain and are considered to be likely pathogenic.

There is no radical therapeutic regimen for ARCI at present, and affected individuals could only undergo limited symptomatic treatment to relieve symptoms. One potentially effective therapeutic strategy including glycolic acid, 10%–20%, cream and a combination cream of lovastatin, 2%, with cholesterol, 2%, has been suggested to yield a satisfactory curative effect with improvement of the cutaneous condition of patients with ARCI (Khalil et al., 2018).

In conclusion, we detected four novel probably disease‐causing mutations in two unrelated nonconsanguineous ARCI families, which expand the mutational spectrum of ARCI type 10, and contribute to genotype–phenotype correlations, and further facilitate the development of genetic counseling of affected families. In addition, this study may laid a solid foundation for the further investigations of ichthyoses pathogenesis and genetic therapy.

## CONFLICT OF INTERESTS

The authors declare no competing interests.
